# Physicochemical characterization of titanium dioxide pigments using various techniques for size determination and asymmetric flow field flow fractionation hyphenated with inductively coupled plasma mass spectrometry

**DOI:** 10.1007/s00216-016-9783-6

**Published:** 2016-07-29

**Authors:** Johannes P. F. G. Helsper, Ruud J. B. Peters, Margaretha E. M. van Bemmel, Zahira E. Herrera Rivera, Stephan Wagner, Frank von der Kammer, Peter C. Tromp, Thilo Hofmann, Stefan Weigel

**Affiliations:** 1 RIKILT Wageningen UR, Akkermaalsbos 2, 6708 WB Wageningen, The Netherlands; 2 Department of Environmental Geosciences, University of Vienna, Althanstrasse 14, UZA II, 1090 Vienna, Austria; 3 Department Analytik, Helmholtz Zentrum für Umweltforschung-UFZ, Permoserstrasse 15, 04318 Leipzig, Germany; 4 TNO Earth, Life and Social Sciences, Princetonlaan 6, 3584 CB Utrecht, The Netherlands; 5 Bundesinstitut für Risikobewertung (BfR), Max-Dohrn-Straβe 8-10, 10589 Berlin, Germany

**Keywords:** Asymmetric flow field flow fractionation, Inductively coupled plasma mass spectrometry, Nanomaterials, Size distribution, Titanium dioxide, Zirconium

## Abstract

Seven commercial titanium dioxide pigments and two other well-defined TiO_2_ materials (TiMs) were physicochemically characterised using asymmetric flow field flow fractionation (aF4) for separation, various techniques to determine size distribution and inductively coupled plasma mass spectrometry (ICPMS) for chemical characterization. The aF4-ICPMS conditions were optimised and validated for linearity, limit of detection, recovery, repeatability and reproducibility, all indicating good performance. Multi-element detection with aF4-ICPMS showed that some commercial pigments contained zirconium co-eluting with titanium in aF4. The other two TiMs, NM103 and NM104, contained aluminium as integral part of the titanium peak eluting in aF4. The materials were characterised using various size determination techniques: retention time in aF4, aF4 hyphenated with multi-angle laser light spectrometry (MALS), single particle ICPMS (spICPMS), scanning electron microscopy (SEM) and particle tracking analysis (PTA). PTA appeared inappropriate. For the other techniques, size distribution patterns were quite similar, i.e. high polydispersity with diameters from 20 to >700 nm, a modal peak between 200 and 500 nm and a shoulder at 600 nm. Number-based size distribution techniques as spICPMS and SEM showed smaller modal diameters than aF4-UV, from which mass-based diameters are calculated. With aF4-MALS calculated, light-scattering-based “diameters of gyration” (Øg) are similar to hydrodynamic diameters (Øh) from aF4-UV analyses and diameters observed with SEM, but much larger than with spICPMS. A Øg/Øh ratio of about 1 indicates that the TiMs are oblate spheres or fractal aggregates. SEM observations confirm the latter structure. The rationale for differences in modal peak diameter is discussed.

## Introduction

Engineered nanomaterials (ENMs) are used in an increasing number of commercial applications varying from photovoltaic cells, pharmaceutical drugs, food additives, cosmetics and others [1]. Titanium dioxide (TiO_2_) has, together with carbon black, carbon nanotubes and silica the largest production volume and a very wide array of applications [2, 3]. Titanium dioxide materials (TiMs) are used mostly as whitener in paints but also in food, as sunscreen blocker in cosmetics, as antibiotic and photocatalyst [1–3].

Despite the widespread use in consumer products, relatively little research has been performed on the characterization of TiMs. Important parameters for biological and chemical activities of NMs and related materials in general are size or size distribution of particles and the presence of additional chemical components [1, 4, 5]. The latter characteristic may or may not be restricted to the surface of the material. Analyses of TiMs were performed on sunscreen blockers, where they are added because of their capacity to absorb and reflect light, especially UV-A and UV-B. This characteristic is very much dependent on particle size distribution [6–9]. These studies describe analytical protocols including field flow fractionation (FFF) for separation, a variety of techniques to determine size distribution and inductively coupled plasma mass spectrometry (ICPMS) or ICP atomic emission spectroscopy (ICPAES) for quantitative measurement of titanium. Titanium dioxide is approved by the European Union (EU) legislation in personal care products in concentrations up to 25 % (*w*/*w*) [10]. For food applications, the EU has approved TiO_2_ as the food additive “E171” as a bulk material. Recently, size distributions of food-grade TiMs and in food products have been described, and the results showed that at least part of this material is in the nano-size range [11, 12]. Some TiMs contain other metal oxides, e.g. Al_2_O_3_, SiO_2_ and ZrO_2_ [8, 9, 13] which may influence their chemical and biological activities. Since ICPMS is capable of measuring more than one metal species in a single run [4, 8], a combination of FFF and ICPMS is very promising to characterize size distribution as well as multi-element composition of TiMs.

The EU published a recommendation for the definition of the term nanomaterial [14] which formulates a “nanomaterial” in the strict sense as “a natural, incidental or manufactured material containing particles, in an unbound state or as an aggregate or as an agglomerate and where, for 50 % or more of the particles in the number size distribution, one or more external dimensions is in the size range 1–100 nm”. In addition, this EU recommendation gives an extension of the definition stating that “In specific cases and where warranted by concerns for the environment, health, safety or competitiveness the number size distribution threshold of 50 % may be replaced by a threshold between 1 and 50 %”. In a recent paper of our laboratory [11], the pigments, which are investigated further in the present study, were shown to contain approximately 10 % of the particles with a diameter in the range of 1–100 nm and the remainder between 100 and 1000 nm. The term “pigments” is used throughout the manuscript to describe our materials.

**Fig. 1:**
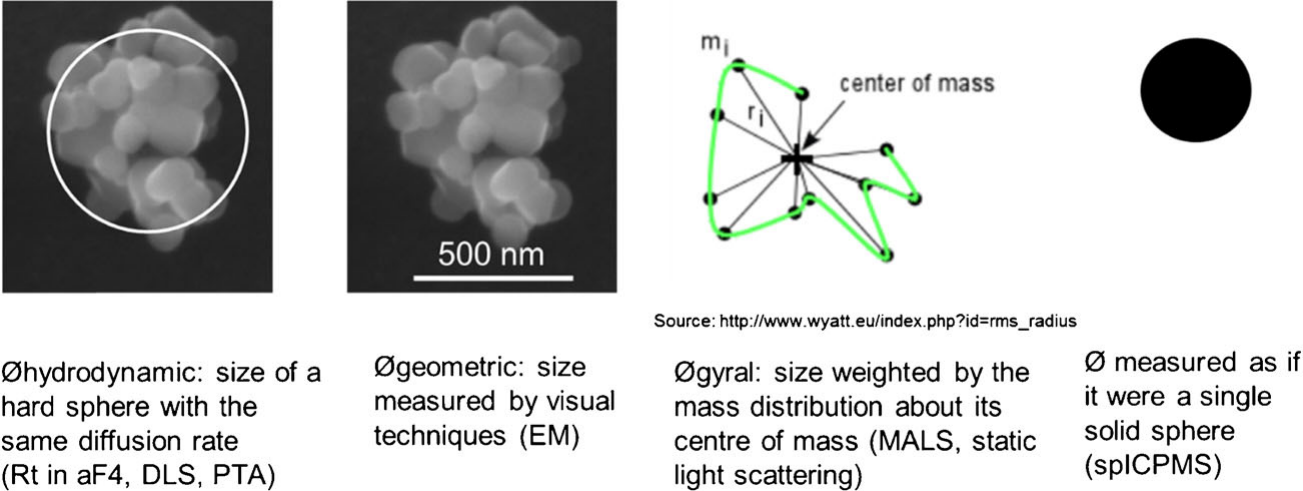
Schematic illustration of different concepts for defining the diameter (Ø = 2 × radius) of nanomaterials. Between brackets the physicochemical techniques which provide these parameters

Relevant particle size parameters that can be distinguished (Fig. 1) are hydrodynamic radius, based on the diffusion rate in aqueous solvents; radius of gyration or root-mean-square radius, which is defined as “mass distribution about its centre of gravity”; and the geometric radius, based on visual measurements. The hydrodynamic radius can be determined using techniques as dynamic light scattering (DLS), FFF fractionation and particle tracking analysis (PTA). The radius of gyration is measured by multi-angle light scattering (MALS). The geometric radius is determined by transmission or scanning electron microscopy (TEM or SEM). Finally, in single particle ICPMS (spICPMS), a sphere-equivalent radius is calculated for individual particles or aggregates thereof based on the measured elemental mass (see below).

**Fig. 2:**
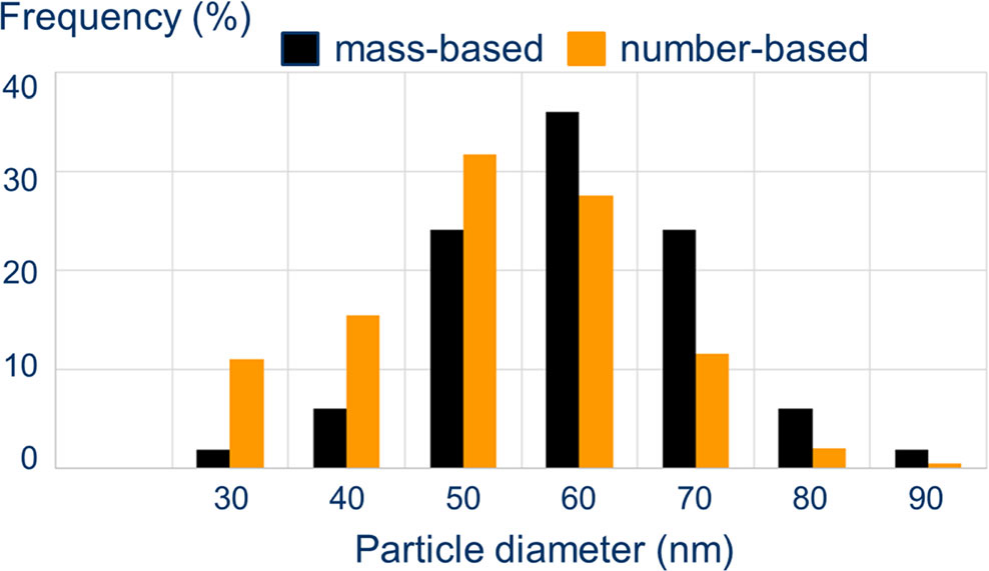
Theoretical model of interconversion between mass- and number-based particle size distributions of the same particle population. Note the 50 nm ↔ 60 nm shift for the modal peak between the two types of size distribution. This is due to the third power relationship between mass (*m*) and radius (*r*) of a particle following the equation *V* = $\frac{m}{\uprho}=\frac{4}{3}\pi\ {r}^3$, where *V* = volume and *ρ* = specific density of a spherical particle

Apart from the method of particle size measurement, the basis for quantification should be considered. Here, three relevant modes can be distinguished: mass-based, number-based and light-scattering intensity-based. Mass-based quantification is observed with ICPMS detection, number-based quantification is realised with electron microscopy, spICPMS and PTA and light-scattering intensity-based quantification results from MALS detection at 0° angle. Quantification by UV or visible light absorption is not only mass-based but also includes a mostly undefined scattering component [7, 15, 16]. Because the radius of a spherical particle and its mass show a third power relationship, a number-based size distribution will show a lower modal diameter than a mass-based size distribution, because the contribution of small particles weighs more strongly [17] (see also Fig. 2). Since at constant number concentrations light scattering has a linear, positive sixth power relationship to radius and a third power relationship to mass, light-scattering-based size distributions will be different from number-based distribution. Mass (-) and light-scattering-based size distributions will be similar because the increase of scattering intensity by nanoparticles with a certain mass will be compensated for by an equivalent decrease in number of particles of that mass.

Single particle ICPMS is a specific mode of ICPMS operation, which enables determination of size of individual (“single”) particles. This technique has been described for silver nanoparticles [18–20] and TiMs [11, 21]. In spICPMS, the particle size is calculated as a sphere-equivalent diameter directly from the measured particle elemental mass and specific density via the equation given in Fig. 2. This calculation will give a particle diameter which assumes the mass as representing a massive, single solid sphere (Fig. 1 and [22]).

In the present study, the major topic is to compare various techniques for size determination either or not in combination with asymmetric flow field flow fractionation (aF4) for separation on nine TiMs. In addition, we investigated the applicability of aF4 hyphenated with ICPMS to establish whether additional elements in the TiMs form an integral part of the particulate material which is separated from low molecular weight and large aggregates (>1 μm) during the elution stage in aF4. Finally, we validated the analytical protocol for aF4 separation in combination with ICPMS quantification for a number of validation parameters to establish analytical performance. Validation has until now rarely been reported for chemical analyses of TiMs and related materials, and if so for only a few validation parameters. We made a comparison between various techniques for measuring particle size, e.g. SEM, spICPMS, MALS and size measurement on the basis of retention time in aF4. Electron microscopy and spICPMS provide number-based size information via metric standards, while MALS shows size distribution with light scattering intensity as quantitative basis. Attempts to determine size distribution with PTA appeared inappropriate for these TiMs (see “Results” section). Seven from the nine TiMs used in this study are food-grade pigments, supplied by commercial suppliers. The other two, NM103 and NM104, are relatively well-defined TiMs and have been described to contain aluminium and silicon as coating materials in the form of Al_2_O_3_ and SiO_2_, respectively, in addition to titanium [8, 13, 23].

## Materials and methods

### Materials

All chemicals were reagent grade. Ultrapure water (resistivity 18.2 MΩ cm^−1^ at ambient temperature) was prepared by a Milli-Q Advantage A10 Water Purification System (Millipore, Amsterdam, Netherlands). FL-70 detergent was purchased from Fischer Scientific (Landsmeer, Netherlands). As stated by the supplier, FL-70 contains tetrasodium ethylenediamineteraacetate 1.4 %, sodium oleate 0.5 %, sodium bicarbonate 0.1 %, sodium carbonate 2.7 %, triethanolamine oleate 3.8 %, water 88.8 %, polyethylene glycol 0.9 %, alcohol, C12-14-secondary, ethoxylated 1.8 % and is free of silicates, phosphates and chromates. Bovine serum albumin (BSA) was purchased from Sigma (Code A-7888). Nitric acid (67–69 %, Instra-Analysed Plus), used to prepare ICP standard solutions, was from J.T. Baker (Avantor Performance Materials B.V., Deventer, Netherlands) and ICP standards from Merck (Darmstadt, Germany). Representative TiMs NM103 and NM104 were a kind gift from the Joined Research Centre (JRC nanomaterials repository, ISPRA, Italy) and have extensively been described by the suppliers [13] and in other publications [8, 23]. The other seven, which have been used in an earlier study [11], are food-grade TiMs provided by commercial suppliers. They are coded Pigment-1 through Pigment-7 in this study. Scanning electron microscopy (SEM) was performed with a TESCAN MIRA-LMH FEG scanning electron microscope (TESCAN Benelux, Brussels, Belgium).

### Compliance with ethical standards

The research did not involve human participants and/or animals.

### Dispersion protocol for nanomaterials

The procedure for dispersion of TiMs in 0.05 % BSA in water has been described before [24]. Shortly, a sample of 15.36 ± 0.10 mg powdered TiM is weighed in a 30-mL sample vial (diameter 24 mm). Thirty microliters of 96 % ethanol is added and distributed equally over the TiM powder. Then, 970 μL 0.05 % BSA solution is added, and the mixture is shaken manually to achieve an even suspension. Another 5 mL 0.05 % BSA is added, and the suspension is shaken to homogeneity. This suspension is exposed to ultrasonic treatment with a needle probe for 16 min at 4 W and 22.5 kHz using a Misonix XL-2000 sonicator with a CML-4 probe (Qsonica, Newton, CT, USA) while the sample vial is cooled in ice water. The final dispersion is analysed after dilution in 0.05 % BSA to the desired TiM concentration. TiM suspensions were prepared daily, analysed within 1 h after preparation and shaken on a Vortex shortly before analysis. BSA is applied as a dispersing agent, not as a matrix component. When BSA was omitted under these relatively mild ultrasound conditions, very fast precipitation was observed and all titanium eluted at the void peak in aF4-ICPMS. Earlier investigations showed good stabilising capacity of BSA on dispersions of similar materials containing silver [25, 26] and also TiO_2_ [27] as the major component with only a small or no effect on the size distribution pattern.

### Validation protocol

For validation studies, TiM concentrations were 10 mg L^−1^, unless otherwise mentioned. For these experiments, asymmetric flow field flow fractionation (aF4) was performed using a 350-μm spacer and a regenerated cellulose separation membrane (see below under “Asymmetric flow field flow fractionation (aF4)” section). Repeatability for aF4-ICPMS analyses was determined as the average of relative standard deviations (STDrel) in peak area for the ^48^Ti-isotope from three series, each including six repeats. For each repeat, a new TiM sample was weighed out and prepared as described above. The three series were performed on different days. The first repeat of each series was excluded from analysis, since it was consistently lower than the subsequent 5, probably due to an unsaturated state of the aF4 membrane. Data are expressed as the ratio $\frac{\mathrm{peak}\ \mathrm{area}\ \mathrm{o}\mathrm{f}\ \mathrm{nanomaterial}\ \mathrm{f}\mathrm{o}\mathrm{r}\ \mathrm{the}\ \mathrm{T}\mathrm{i}-\mathrm{isotope}}{\mathrm{Rh}-\mathrm{signal}\ \mathrm{i}\mathrm{ntensity}\ \mathrm{o}\mathrm{f}\ \mathrm{i}\mathrm{nternal}\ \mathrm{standard}\kern0.37em \left(\mathrm{see}\ \mathrm{below}\right)}$. Reproducibility was defined as the overall, relative standard deviation from the 15 analyses in 3 series. Standard deviations were calculated using ANOVA.

### Asymmetric flow field flow fractionation (aF4)

Two different aF4 equipment types were used for flow control because they were performed at different locations: for aF4 with UV and ICPMS detection performed at RIKILT, Wageningen UR, Wageningen (Netherlands) flow control was provided by an Eclipse Dualtec separation system (Wyatt Technology Europe, GmbH, Dernbach, Germany), while for aF4-MALS measurements at the University of Vienna, Vienna (Austria) an Eclipse 3+ system was used. Separation was performed using a 350-μm or 250-μm spacer in a short channel flow cell with dimensions 153 × 22 mm, containing a Nadir regenerated cellulose (RC) or polyethersulfon (PES) separation membrane with 10 kDa MWCO (Wyatt Technology Europe). Carrier solvent was an aqueous solution of FL-70 (Fischer Scientific) and NaN_3_ (both 200 mg L^−1^, 3.08 mM for NaN_3_) which was filtered before use over 0.45 μm-HA filters (Millipore, Amsterdam, Netherlands) to remove particulate materials. The aF4 system was further equipped with an HPLC pump for solvent flow delivery, degasser and autosampler, all of the Agilent 1100 series (Agilent Technologies, Amsterdam, Netherlands). Detection was performed with a Knauer K-2600 UV-detector set at 254 nm and/or ICPMS (see below). Samples of 10 or 50 μL were injected and subjected to the following flow protocol:

**Table:** 

Time (min)	Description	Detector flow (mL min^−1^)	Focus flow	Cross flow	Injection flow
0–2	Elution	0.5	0	0	0
2–3	Focus	0.5	1.5	0	0
3–5	Focus + injection	0.5	1.5	0	0.2
5–7	Focus	0.5	1.5	0	0
7–57	Elution	0.5	0	0.1	0
57–62	Elution	0.5	0	0	0
62–67	Elution + injection	0.5	0	0	0.2
67–69	Elution	0.5	0	0	0

Under these conditions, size calibration was performed using polystyrene standards of defined sizes: 20 nm, 30 nm, 40 nm, 46 nm, 102 nm, 203 nm, 350 nm and 700 nm from Duke Scientific (Palo Alto, CA, USA, purchased via Distrilab, Leusden, Netherlands) dispersed in 0.05 % BSA. Retention times were measured by UV detection at 254 nm. For aF4-UV analyses, TiMs were diluted to 250 mg L^−1^ from which 10 μL was injected.

Recovery of analytes was measured as percentage of peak area relative to that obtained with a aF4-protocol, where no focussing flow or cross flow were applied:

**Table:** 

Time (min)	Description	Detector flow (mL min-1)	Focus flow	Cross flow	Injection flow
0–1	Elution	0.5	0	0	0
1–11	Elution + injection	0.5	0	0	0.2
11–12	Elution	0.5	0	0	0

### Inductively coupled plasma mass spectrometry

**Fig. 3:**
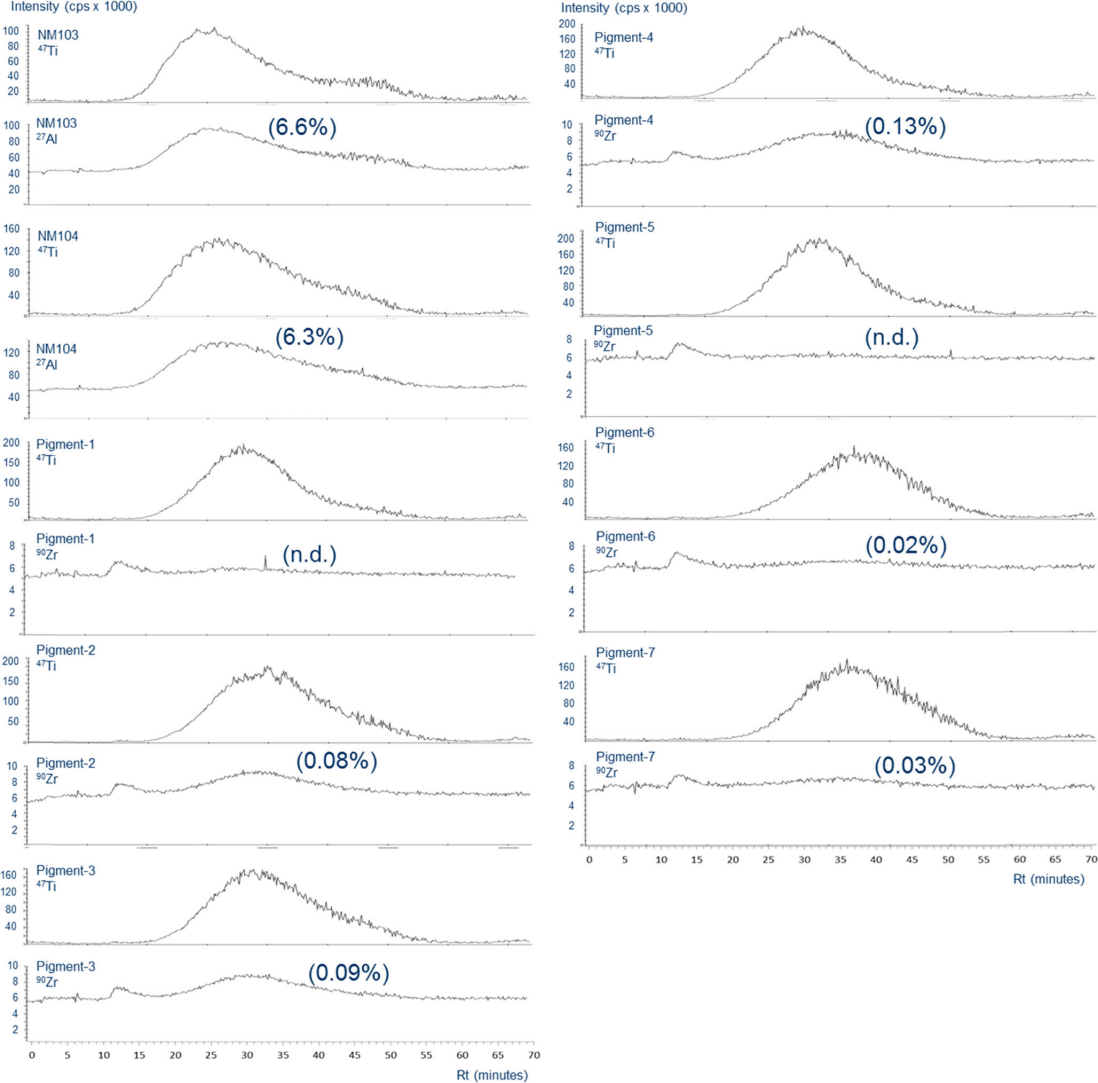
Asymmetric flow field flow fractograms with multi-element ICPMS detection of nine TiO_2_ materials. Between brackets the mass proportions of elements other than TiO_2_ in the aF4 peak of the materials. The proportion of aluminium is given as Al_2_O_3_ and that of zirconium as ZrO_2_. *n.d*. not detectable

After aF4 separation of the TiMs, inductively coupled plasma mass spectrometry (ICPMS) analyses were performed online on a Thermo X Series 2 ICPMS, equipped with a Burgener PEEK Mira Mist type nebulizer and quartz impact bead spray chamber. The ICPMS settings were optimised daily, and the equipment was operated at an RF power of 1400 W. Titanium was measured as the ^48^Ti-isotope for validation experiments (Table 1) to achieve maximal sensitivity and as the ^47^Ti-isotope for multi-element detection (Fig. 3). The ^48^Ti-isotope has a natural abundance of 73.7 % and hence a much higher signal-to-noise ratio than the less abundant ^47^Ti-isotope (7.4 %). Use of the ^47^Ti-isotope allows a much higher TiM concentration of 100 mg L^−1^ in the sample and thus leads to a lower limit of detection for the other elements. At 100 mg L^−1^, the ICPMS detector was saturated for the ^48^Ti-signal. ^44^Ca was measured to correct for any ^48^Ca interference on the ^48^Ti-signal. ^103^Rh was used as internal standard for instrumental drift and supplied as RhN0_3_ at 20 μg Rh L^−1^ solution in 2.8 % HNO_3_. It was continuously infused with a peristaltic pump via a T-piece directly into the ICPMS nebulizer at a flow rate of 0.75 mL min^−1^. Aluminium, silicon and zirconium were measured as ^27^Al-, ^28^Si- and ^90^Zr-isotopes, respectively. The dwell time for all isotopes was 1 s. Quantification was based on peak area and calibration lines were determined by injecting fixed amounts of ICP standards (Merck KGaA, Darmstadt, Germany) into the ICPMS nebulizer via the T-piece. During these analyses, the aF4 cell flow was maintained at 0.5 mL min^−1^. Measured contents for Ti, Al, Si and Zr are converted to TiO_2_, Al_2_O_3_, SiO_2_ and ZrO_2_ by correction for the presence of oxygen.

**Table 1:** Validation of FFF-ICPMS analyses for 2 TiO_2_ materials: NM104 and Pigment-7. *V*x = cross flow (mL min^−1^)

TiO_2_ type	NM104	Pigment-7
Repeatability within three series of five repeats (relative standard deviation in percent of average)	7.8	3.9
Reproducibility between three series (relative standard deviation in percent of average)	9.7	11.7
Dynamic range (mg L^−1^)	0.1–70 Detector saturated at 100 mg L^–1^	1–100
Linearity (*R* ^2^)	0.999	0.994 (0.999 from 1 to 70 mg L^−1^)
Limit of detection (mg L^−1^)	0.05	0.5
Recovery as peak area ratio at *V*x = 0.1/*V*x = 0 (%)	82 ± 11	97 ± 3

### Single particle ICPMS

The protocol for single particle ICPMS (spICPMS) has been elaborately described in [11]. Shortly, TiM suspensions of 50 μg L^−1^ were injected into the ICPMS equipment at 0.5 mL min^−1^ using a peristaltic pump. The ICPMS was operated in time-resolved analysis (TRA) mode with a dwell time of 3 ms and an acquisition time of 60 s per measurement with ^48^Ti as the target isotope. The nebulising efficiency was calculated using a 60-nm gold nanoparticle suspension (code SRM8013, purchased from NIST, Gaithersburg, MD, USA) at 50 ng L^−1^. For conversion of the spICPMS signals into sphere-equivalent particle diameters, an in-house developed, calculation spreadsheet was used. This spreadsheet and a procedure to perform spICPMS analysis have been described earlier [22]. The spreadsheet converts the measured Ti mass into the equivalent TiO_2_ mass by multiplication with 1.457.

### Multi-angle laser light spectrometry

aF4 was hyphenated with a multi-angle laser light spectrometry (MALS) detector for determination of diameters of gyration of the fractionated TiMs dissolved at 250 mg L^−1^ to achieve appropriate signal intensity. The MALS detector (DAWN® HELEOS™, Wyatt Technology Europe GmbH, Dernbach, Germany) was operated with 17 + 1 observation angles and a linearly polarized laser beam at λ = 658 nm. Data acquisition was set at 2 s. The collected light scattering response was processed using ASTRA software (Wyatt, Dernbach, Germany) to calculate diameters of gyration (Øg), also known as root-mean-square diameter (= 2 × root-mean-square radius, r_rms_). For the nine TiMs investigated, fitting models, provided with the ASTRA software, were evaluated on three criteria: (1) pre-existing information on particle shape; (2) the calculated ratio of Øg/Øh, which gives indications on the particle shape. (3) It is expected that particle size increases linearly with retention time during aF4 fractionation. If this is indicated by the calculated Øg data, the selected model is able to appropriately reflect the particle elution behaviour. In the present study, all models were tested and the Berry model was selected according to the listed criteria.

### Particle tracking analysis

For particle tracking analyses, NanoSight LM20 equipment (NanoSight Ltd., Wiltshire, UK) was used. TiMs were investigated at various concentrations ranging from 25.6 to 2560 mg L^−1^. Dilution was carried out with water and 0.05 % BSA. Instrumental settings, e.g. detection threshold, shutter time, screen gain, blur, minimum expected particle size, camera frame rate, track length and bin width, were varied as described by the supplier. Data were developed using NTA 2.1 software.

### Electron microscopy

For determination of size distribution of the TiM, suspensions at 2.56 mg mL^−1^ were analysed according to [11]. Briefly, suspensions were filtered over an Anopore aluminium oxide filter (20 nm pore size) to retain the TiMs and avoid the formation of agglomerates that can result from droplet drying. Filters were mounted on aluminium specimen holders, coated with a 5-nm layer of chromium and analysed using a TESCAN MIRA-LMH FEG SEM at an accelerating voltage of 15 kV. Data were processed with the Scandium SIS software package (Olympus Soft Imaging Solutions, Germany). To measure size distribution for particles from 25 to 1600 nm, three magnifications were selected: ×10,000, ×25,000 and ×75,000 and particles were scored in the following size bins: 25–40 nm, 40–65 nm, 65–100 nm, 100–160 nm, 160–250 nm, 250–400 nm, 400–650 nm, 650–1000 nm and 1000–1600 nm. At least 10 particles per size bin and in total 1000 particles were taken for each TiM type. The size range of primary particles in the TiMs was determined manually in this study from the electron micrographs by measuring approximately 500 particles.

## Results

### Sample preparation and optimisation of aF4-ICPMS separation performance

**Fig. 4:**
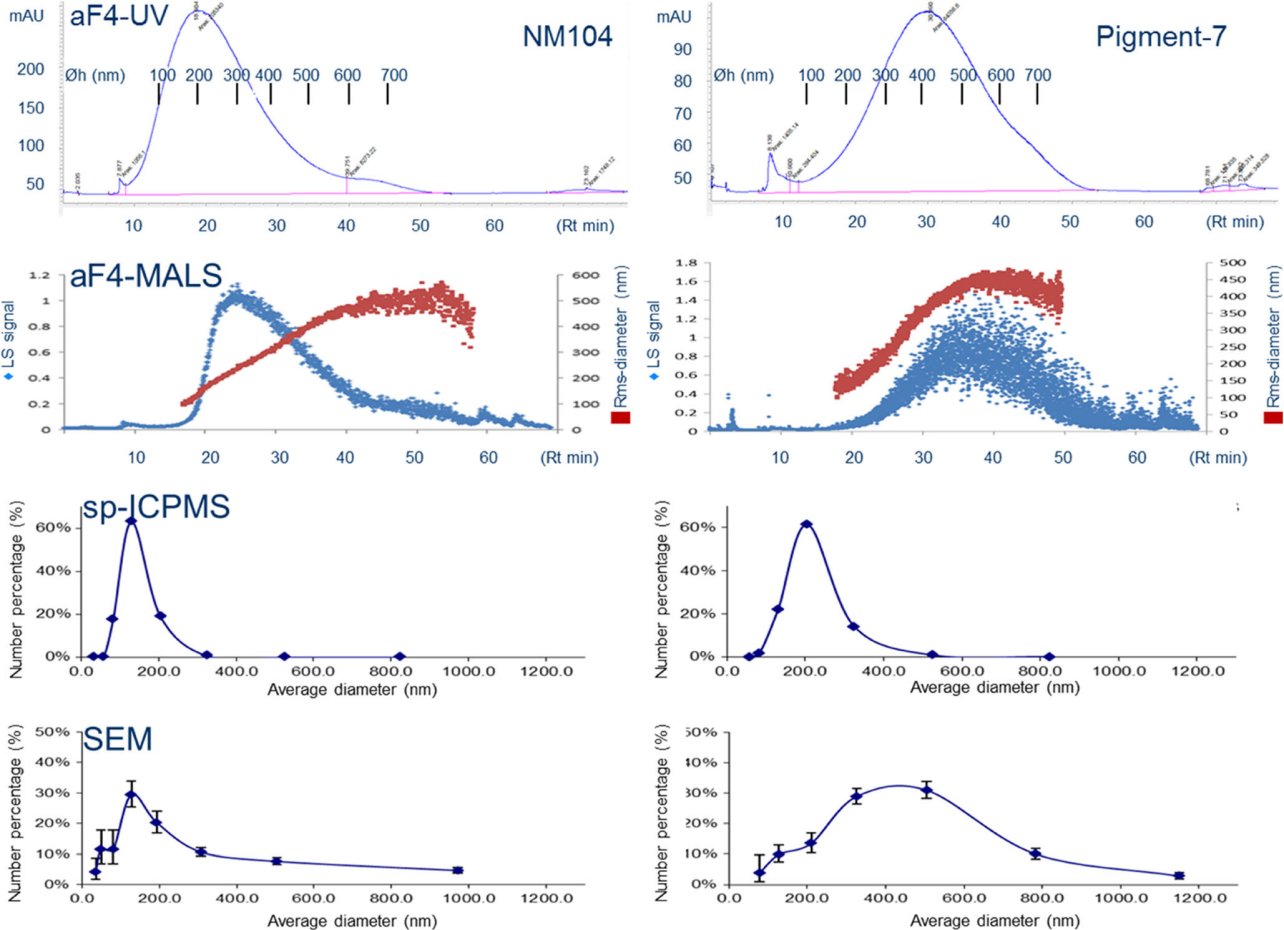
Comparison of various techniques for size measurement of TiO_2_ materials, types NM104 and Pigment-7. *aF4-UV* asymmetric flow field flow fractionation combined with UV detection at λ = 254 nm and calibration with polystyrene nanoparticles of defined size, *aF4-MALS* asymmetric flow field flow fractionation combined with multi-angle laser light spectrometry, *spICPMS* single particle inductively coupled plasma mass spectrometry, *SEM* scanning electron microscopy. Hydrodynamic diameters (Ø_h_ in nm), as indicated by *bars* for aF4-UV analyses in the top two diagrams, are calculated from a calibration line obtained with polystyrene nanoparticles of defined size. Numeric results for size determination for all nine TiO_2_ materials investigated with the four techniques are given in Table 2

Regenerated cellulose (RC) membranes were chosen for aF4 separation, since they gave sharper peaks than polyether sulfone (PES) membranes. Moreover, using RC membranes, less material eluted in the release peak, where large nanoparticles (>700 nm) elute after the cross flow (Vx) is decreased from 0.1 to 0 mL min^−1^. An aF4 cell spacer height of 350 μm resulted in less TiM eluting in the release peak as compared to a spacer height of 250 μm. At an elution time of about 50 min, with a cross flow of 0.1 mL min^−1^, virtually all TiO_2_ eluted before the release peak, while little TiO_2_ was observed in the void peak where small (<5 nm) particles would elute (Figs. 3 and 4). Considering this information, the conditions optimised for aF4 analyses of the nine TiM types are as follows: a continuous cross flow of 0.1 mL min^−1^, regenerated cellulose with a MWCO of 10 kDa as separation membrane and a spacer height of 350 μm. On the basis of literature sources, a carrier solvent containing FL-70 and NaN_3_ (both at 200 mg L^−1^) was chosen for the nine TiM types [6–8].

### Validation of the FFF-ICPMS analytical protocol

The analytical protocol as applied for aF4-ICPMS characterization of the TiMs was subjected to various parameters for validation of analytical chemical protocols for the smallest hydrophilic type, NM104, and largest TiM type, Pigment-7 (Table 1). Limits of detection were 0.05 and 0.5 mg L^−1^ for these two TiM types, respectively, when measured with the ^48^Ti-isotope in ICPMS. Linearity within a dynamic range from 0.1 to 70 mg L^−1^ for NM104 and from 1 to 100 mg L^−1^ for Pigment-7 was good with *R*^2^ values of 0.999. Despite a peak width of more than 10 min and a high noise level at the end of the peak (Fig. 3), repeatability values of 4–8 % (relative standard deviation) and reproducibility values of about 10 % are regarded as acceptable for analyses of nano- and related materials. Recoveries, determined as percentage of peak area obtained when no cross flow was applied, were 82 ± 11 % for NM104 and 97 ± 3 % for Pigment-7.

### Multi-element composition of TiO_2_ materials

Figure 3 illustrates that besides titanium, NM103 and NM104 also contain aluminium which co-elutes with the titanium peak between the void and release peaks. The same is observed for zirconium in five out of seven commercial pigments. Co-elution of aluminium and zirconium with titanium indicates that they form an integral part of the TiM structure. Aluminium oxide was observed at proportions of total metal content of 6.6 and 6.3 %, respectively, the remainder being titanium dioxide. Zirconium oxide concentrations were measured in the range of 0.02–0.13 % of total metal species. In addition, all seven commercial pigments showed a small zirconium peak at the void volume of the fractogram which forms about 0.02 % of total metal species (Fig. 3). Silicon was not detected in any of the nine TiMs.

### Comparison of size determination techniques for nine TiO_2_ materials

PTA appeared inappropriate for size determination of the TiMs investigated since the observed size distribution for a single sample varied strongly with the instrument settings, especially with shutter time and gain. These two PTA settings are dependent on the size of the nanoparticles. The strong bias of light scattering intensity towards larger particles (see “Discussion” section) is very likely the reason for the inappropriateness of PTA for analysis for the polydisperse TiM populations. Very small particles will be completely and medium size particles partially excluded from the particle size distribution analysis. The exclusion rate can be manipulated by PTA equipment settings, but in this way it is not possible to obtain unequivocal size information.

The TiM types investigated showed similar patterns for all four size determination techniques including SEM. This is illustrated in Fig. 4 for TiM types NM104 and Pigment-7 and is also evident from aF4-ICPMS analyses of all nine materials (Fig. 3). A modal peak is observed in the range between 200 to 500 nm with aF4-ICPMS, aF4-UV, aF4-MALS and SEM detection and at lower values with spICPMS. The first four size determination techniques show a more or less pronounced shoulder for larger diameters.

A common technique for size determination of NMs, requiring relatively little sophisticated equipment, is to relate their retention time after aF4 separation with those of commercially available standards of defined diameter, e.g. polystyrene nanoparticles. Detection can be performed with either ICPMS or UV-absorption (Fig. 4 top panels, Table 2). UV-absorption was chosen for size measurement in our studies because it can detect both the calibrant polystyrene nanoparticles and the analyte TiMs. The retention time versus diameter regression in our study was linear with *R*^2^ > 0.99 for polystyrene particles from 20 to 350 nm and >0.98 when 700 nm NPs are also included (data not shown). According to FFF theory, this linearity is expected when the cross flow in aF4 is kept constant. Table 2 shows the modal hydrodynamic diameters (Øh) values of all nine TiMs after aF4-UV and the other three size determination techniques. TiM concentrations up to 1000 mg L^−1^ did not influence peak retention time in aF4-UV and thus did not have impact on calculated hydrodynamic diameters. The TiM types NM103 and NM104 are the smallest with modal diameters of approximately 200 nm and Pigment-6 and Pigment-7 are the largest with modal diameters over 400 nm. The ranking order of size distribution of hydrodynamic diameters (Øh) between the nine TiMs is similar to that obtained with MALS measurements for diameters of gyration (Øg) which was also performed in combination with aF4 separation. According to the selection criteria, the rms radii were calculated by the Berry model which assumes an arbitrary shape of the particle. Although the Berry model gives erroneous results for larger particles (e.g. Øg > 220 nm for spherical particles) [28], it was the most suitable for most investigated samples. However, at elution time of 35 to 40 min (or >450 nm), the Berry model underestimates the particle size. Both Debye and Zimm model produced non-linear increasing particle sizes and unlikely Øg/Øh ratios (<0.775). Therefore, they were excluded for data analysis. The Øg/Øh ratios as given in Table 2 vary from 0.89 to 1.09 with an outlier to 1.26 for Pigment-1. Calculated over all nine TiMs tested, the Øg/Øh ratios in Table 2 were 1.02 ± 0.12.

**Table 2:** Size determination of nine TiO_2_ materials, from which two are reference materials, NM103 and NM104, and seven are pigments provided by commercial suppliers

TiO_2_ material	Rt in aF4 (min)	aF4-UV (Øh) (nm)	aF4-MALS (Øg) (nm)	Øg/Øh ratio	spICPMS (nm)	SEM aggregate size bin (modal) (nm)	Primary particle (nm)
NM103	9.8	209	190	0.91	122	100–160 (155)	13–35
NM104	10.6	224	240	1.07	120	100–160 (135)	13–35
Pigment-1	15.7	333	420	1.26	128	160–250 (200)	60–300
Pigment-2	16.2	343	370	1.08	159	250–400 (320)	60–300
Pigment-3	16.7	354	370	1.05	221	250–400 (320)	60–300
Pigment-4	17.0	360	310	0.86	156	250–400 (320)	60–300
Pigment-5	17.4	368	400	1.09	220	250–400 (345)	60–300
Pigment-6	19.5	414	390	0.94	156	250–400 (375)	60–300
Pigment-7	22.7	480	440	0.92	158	400–650 (415)	60–300

Size determinations by asymmetric flow field flow fractionation (aF4) separation and UV_254 nm_ detection were performed on the basis of retention time after calibration with polystyrene standards. *spICPMS* single particle ICPMS, *SEM* scanning electron microscopy, *Øg* gyral diameter in nm from aF4-MALS detection using the Berry model for data processing, *Øh* hydrodynamic diameter in nm. SEM results are given as size bins in which modal diameters are observed and as size range (smallest-largest) in diameters of the constituting primary particles. Between brackets the modal diameters, estimated from a fit of SEM data points

A third approach for studying size distribution is single particle ICPMS (spICPMS), where masses of individual aggregates can be determined at very low concentrations and using very short dwell times in ICPMS detection. Diameters determined by spICPMS were consistently much smaller than with the other three analysis techniques (Table 2). With spICPMS, NM103 and NM104 are again the smallest but now Pigment- 3 and Pigment-5 are the largest materials.

**Fig. 5:**
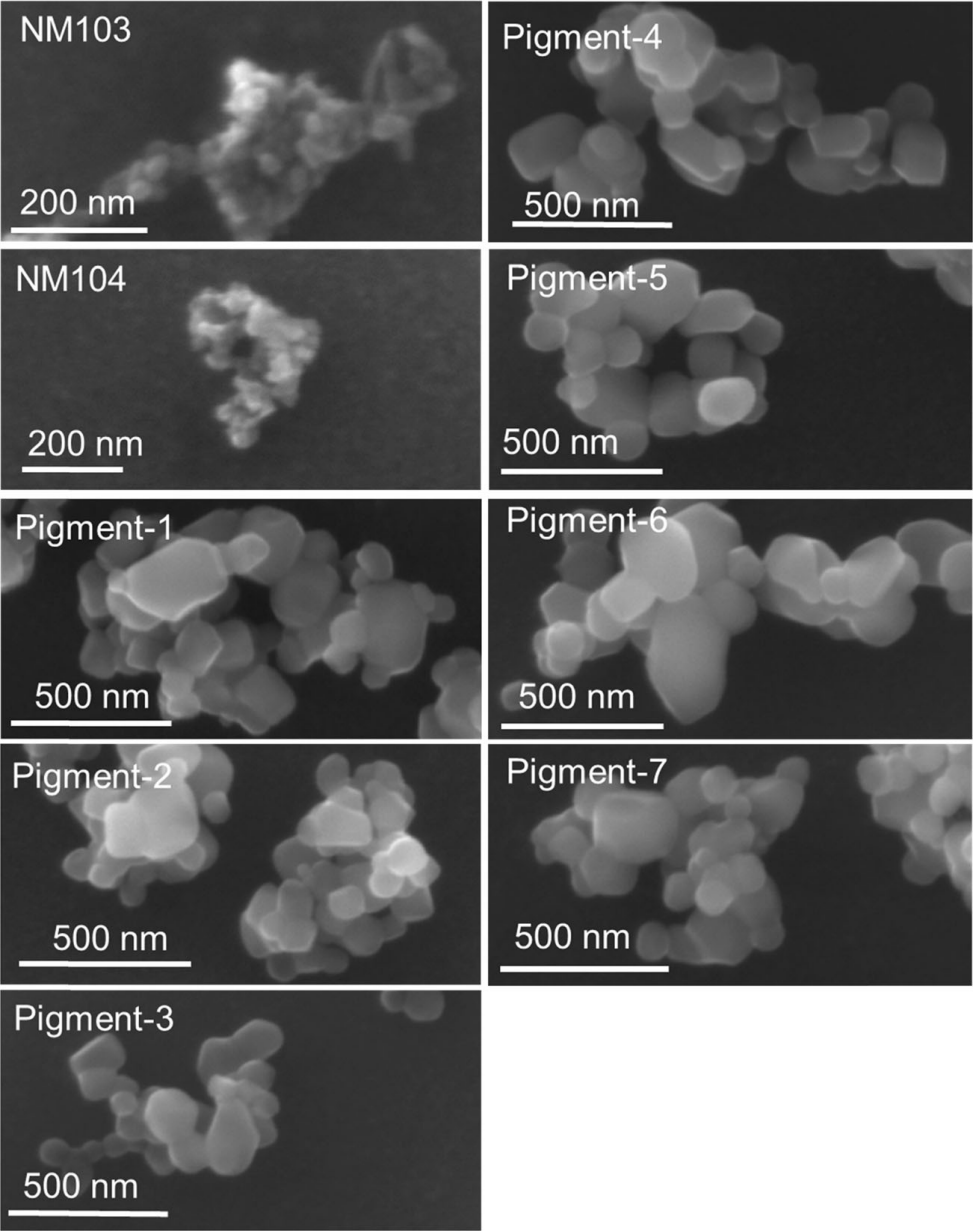
Scanning electron micrographs of nine TiO_2_ materials. Electron micrographs of Pigments 1 and 5 have been published in an earlier study of our group [11] and have been provided with permission of the copyright holder

A very comprehensible way to provide particle size distribution, and therefore by many considered the “golden standard”, is to analyse nanoparticle diameters by electron microscopy. Figure 5 shows that the nine TiMs consist of fractal aggregates. Fractal aggregates are formed by diffusion-limited cluster aggregation or reaction-limited cluster aggregation of primary particles [29]. Sample preparation, e.g. presence of dispersants like BSA and sonication protocols, might affect aggregation state. Therefore, we used a standardized and well-described protocol [24] for comparison between the nine TiMs. The TiMs in the present study are composed of primary particles, which are similar in size at 13–35 nm for NM103 and NM104 and more variable in size in the range from 60 to 300 nm for Pigments 1–7 (Table 2). SEM-based size determinations show NM103 and NM104 as the smallest TiMs with modal geometrical diameters of about 150 nm and Pigment-7 as the largest with a modal diameter of 415 nm. In general, the order in measured diameters between the size determination techniques is aF4-UV>SEM>>spICPMS. For 6 out of 9 TiMs, a larger diameter was measured with SEM as compared to aF4-MALS, while for the other three they are smaller.

From the results in Table 2, it may be concluded that, with the four techniques used for size determination, the ranking order is comparable between the nine TiM types with NM103 and NM104 being the smallest and Pigment-7 the largest TiM type, although there are a few exceptions: (1) Pigment-7 is not the largest TiM type upon spICPMS analysis, and (2) Pigment-3 and Pigment-5 are the largest in spICPMS, but belong to average-sized TiM types in the other three techniques.

## Discussion

The present study describes two parameters relevant for characterisation of polydisperse TiMs: particle size distribution and chemical composition. In addition, a validation is given of the chemical analytical protocol which was most appropriate for this purpose: aF4 for separation and ICPMS for chemical characterization. To our knowledge, this is the first study that provides a comparison between five different techniques for size determination, which forms the major issue in this study, and a method validation of aF4-ICPMS analysis of TiO_2_ particles on six validation parameters for polydisperse materials in the 1–1000 nm size range as the nine TiMs in our study. In an earlier report [11], some of these TiMs were investigated for their total titanium content and for exploration of the use of aF4 with UV detection to establish a number-based size distribution. Polydispersity is a general characteristic for pigment-grade TiMs and a consequence of the various states of aggregation and agglomeration up to micrometer size of primary TiO_2_ nanoparticles with diameter ranging from 5 to 20 nm [17]. In our study, the primary nanoparticle size varied between the materials investigated, 13–35 nm for NM103 and NM104 and 60–300 nm for Pigments 1–7 (Table 2). Particle size distribution analysis of aggregates in the present study includes a comparison of four appropriate techniques for size determination. For the TiMs investigated, sizes of about 200 nm diameter were found for the aluminium-containing species NM103 and NM104. This is in good agreement with earlier observations for these TiMs [17, 23, 30–32]. The commercial pigments investigated in the present study were all larger, varying from 300 to 500 nm in modal hydrodynamic diameter. Size determinations for some of these commercial pigments have been described in an earlier study by our laboratory [11]. Commercial pigments which are allowed as food ingredients by EU and/or US legislations have rarely been investigated before.

Validation of analytical chemical protocols for nano- and related materials has rarely been reported, although the need for this has been mentioned [15]. The studies reporting partial validation mostly concern monodisperse nanoparticles [33–35]. Also for TiMs validation has been reported for rather complex matrices like sun creams and food products [6–8, 11, 27, 32]. The present study describes a more extensive validation of the smallest and largest TiM. The method showed good performance for various validation parameters such as linearity, recovery, limit of detection, reproducibility and repeatability. The results are consistent with the above-mentioned earlier studies on this issue.

The combined use of aF4 for separation and ICPMS or ICP AES for very sensitive and selective detection of elemental species has been described for nanoparticles containing other metals, e.g. gold, silver and silica [33, 34, 36] but also for TiMs [6, 8, 11]. The non-food TiM types NM103 and NM104 have earlier been shown to contain a considerable proportion of aluminium without prior separation of the nanoparticle population [13]. As in our study, simultaneous analysis of more elements after aF4 separation and ICPMS detection has been reported for NM104 [8]. Also for NM103, we observed that aluminium co-eluted with the TiM peak, showing that both elements were an integral part of the TiM structure and not part of smaller or larger units eluting at the void peak (visible at 8 min in ICPMS; Fig. 3) or release peak (at 58 min), respectively. The proportions observed in our study for aluminium in NM103 and NM104, 6.6 and 6.3 % of total metal species, respectively, were close to concentrations of 5.6 and 5.5 % reported by the supplier, who used energy dispersive X-ray spectroscopy for quantification [13]. Silicon, which was reported to be present at more than 100-fold lower concentrations than titanium, was not detected in our study. Five out of seven of the commercial pigment types investigated were observed to contain low concentrations of zirconium co-eluting with the TiM peak in aF4. Zirconium may be present as one the components of the ore material from which the TiMs are prepared [37, 38]. Our study forms the first report of co-occurrence of zirconium as an integral part of TiMs.

Comparison of the various techniques for size determination and in aF4-ICPMS analyses shows similarity in the observation of a broad modal peak in the size range for hydrodynamic diameters from 200 to 500 nm and a shoulder between 600 and 700 nm. Due to better separation, a more pronounced shoulder is anticipated for those TiM types when the major peak is observed at smaller sizes. The fact that this shoulder is also observed in SEM shows that it reflects larger particles and is not due to aberrant aF4 behaviour, e.g. to steric mode instead of Brownian mode separation [15].

Diameter values for TiM aggregates obtained from retention time in aF4 after UV detection are hydrodynamic diameters (Øh), while gyral diameters result from MALS measurement. An interesting phenomenon is that gyral diameters (Øg) are similar to the corresponding hydrodynamic diameters (Øh) given by aF4-UV or aF4-ICPMS detection. In our study, the Øg/Øh ratios vary from 0.86 to 1.26 (Table 2). In polymer chemistry, the Øg/Øh ratio is known as the “shape factor”; it gives an indication of the shape of macromolecules or aggregates. Ratios of about 0.5 are observed for soft spheres, a value of 0.77 for hard spherical particles, values of approximately 1 for oblate spheroids and fractal aggregates, while Øg/Øh ratios >2 are observed for prolate-shaped structures with aspect ratios of more than 1/100 [28, 39–43]. The Øg/Øh ratios of approximately 1.0 (1.02 ± 0.12) observed for the TiMs in our study indicate an oblate spheroid or fractal aggregate shape. A fractal aggregate structure is confirmed by scanning electron microscopy of the same materials (Fig. 5).

Size determination with spICPMS, the third approach used in our study, gives a number-based size distribution where the diameter is calculated from the mass of single aggregates. The measured masses (m) are converted to sphere-equivalent diameters (2 × radius) given the following equation:

$$ m\kern0.5em =\kern0.5em \uprho \kern0.5em 4/3\kern0.5em \uppi \kern0.5em {r}^3. $$

where ρ is the density (4.23 g cm^−3^ for TiO_2_) and 4/3 π *r*^3^ is the volume of a sphere-shaped object. The much smaller size obtained for diameters measured with spICPMS may be expected since size calculation in spICPMS reflects the TiM mass as if it were concentrated in a single solid sphere. The potential impact of not considering the presence of elements other than Ti, which form maximally 6.6 % of the total mass (Fig. 3), is too small to account for the observed differences. The other size determination techniques give hydrodynamic, gyral or geometric diameters which also include the cavities between the TiO_2_ primary unit nanoparticles. As obvious from the SEM pictures in Fig. 5, the packing between the primary nanoparticles in the fractal aggregates is rather loose and thus a two- to threefold smaller particle diameter, calculated from spICPMS data as compared to the other size determination techniques, is readily explained (see also Fig. 1).

In comparing various size determination techniques, it is relevant to consider whether particle quantification is based on numbers, mass or light scattering intensity. As described in the “Introduction” section, there is a third power relationship between number and mass and a sixth power dependence between particle diameter and laser light scattering intensity at angle 0° (I) which follows the equation [44]

$$ I \propto {I}_0\frac{c}{2{r}^2}{\left(\frac{2\pi }{\lambda}\right)}^4{\left(\frac{d}{2}\right)}^6{\left[\frac{m^2-1}{m^2+2}\right]}^2 $$

where *I*_o_ is the intensity of incident laser light, *c* is the number, or number-based concentration of particles, *r* is the distance to the particle, λ is the wavelength of the incident light, *d* is diameter of the particle and *m* is the relative refractive index. Given these relationships, modal diameters obtained with number-based quantification techniques, like SEM and spICPMS, are expected to shift towards smaller values as compared to mass- and/or light scattering-based size distributions, because the same number of smaller particles will have less weight than larger particles in the latter two quantification strategies [17] (see also Fig. 2). ICPMS is a mass-based detection method, MALS quantification is based on light-scattering intensity and UV-absorption is mass-based with also a hard to define component of light-scattering. From the data in Table 2, it is evident that the number-based modal diameters obtained with spICPMS and SEM are smaller than those obtained with mass-based data from aF4-UV, as expected from the above rationale. For aF4-MALS, the calculated gyral diameters are smaller for the three smallest TiMs than with SEM and vice versa for the other six. Very likely, the specific nature of gyral diameters and the rather complex mathematical processing required to calculate it from MALS data do not allow a straightforward quantitative comparison with hydrodynamic or geometric diameters among various materials.

This study shows that the multi-method approach with various size determination techniques and aF4-ICPMS reveals a general pattern for the size distribution of TiMs with one modal peak for hydrodynamic diameter in the 200–500 nm range and a shoulder between 600 and 700 nm. It also demonstrates clear differences between the two TiMs NM103 and NM 104 on one side and the seven commercial pigments tested: the main peak for hydrodynamic diameter in NM103 and NM104 was smaller and contained Al_2_O_3_, in addition to TiO_2_, as integral part of the titanium peak and shoulder. Five out of seven commercial pigments contained zirconium, probably as ZrO_2_, as an additional component. Another difference is the small, relatively uniform size of the primary particles for both NM103 and NM104 and the polydispersity at larger sizes in the commercial pigments. The conclusions are valid for the commercial, food-grade pigments used in the present investigation, but should not generalised to other pigments. This study also indicates that size distributions, obtained with different analytical techniques, are not in all instances accurately interconvertible. This is relevant for defining a material as nanomaterial, as required for legislative purposes by the EU and other governmental authorities. Although labour-intensive, electron microscopy (EM) remains the most generally accepted technique. It is used as the standard technique for defining material as “nano” [1, 23] because it results in a number-based quantification of geometric diameters including the cavities between the primary nanoparticles. Size determination on basis retention time after aF4 separation with either UV, ICPMS or UV detection reasonably approximate these EM-derived diameters and are much less time consuming but they do not directly give number-based quantification. Size distributions obtained with spICPMS, although it provides number-based quantification and is very sensitive [11], should be regarded with considerable care because calculated diameters neglect the cavities between the primary particles.
